# Genomic characterization of five deletions in the LDL receptor gene in Danish Familial Hypercholesterolemic subjects

**DOI:** 10.1186/1471-2350-7-55

**Published:** 2006-06-26

**Authors:** Peter H Nissen, Dorte Damgaard, Anette Stenderup, Gitte G Nielsen, Mogens L Larsen, Ole Færgeman

**Affiliations:** 1 Department of Clinical Biochemistry, Aarhus Sygehus, Aarhus University Hospital, Aarhus, Denmark; 2 Department of Medicine and Cardiology, Aarhus Sygehus, Aarhus University Hospital, Aarhus, Denmark

## Abstract

**Background:**

Familial Hypercholesterolemia is a common autosomal dominantly inherited disease that is most frequently caused by mutations in the gene encoding the receptor for low density lipoproteins (LDLR). Deletions and other major structural rearrangements of the *LDLR *gene account for approximately 5% of the mutations in many populations.

**Methods:**

Five genomic deletions in the LDLR gene were characterized by amplification of mutated alleles and sequencing to identify genomic breakpoints. A diagnostic assay based on duplex PCR for the exon 7 – 8 deletion was developed to discriminate between heterozygotes and normals, and bioinformatic analyses were used to identify interspersed repeats flanking the deletions.

**Results:**

In one case 15 bp had been inserted at the site of the deleted DNA, and, in all five cases, Alu elements flanked the sites where deletions had occurred. An assay developed to discriminate the wildtype and the deletion allele in a simple duplex PCR detected three FH patients as heterozygotes, and two individuals with normal lipid values were detected as normal homozygotes.

**Conclusion:**

The identification of the breakpoints should make it possible to develop specific tests for these mutations, and the data provide further evidence for the role of *Alu *repeats in intragenic deletions.

## Background

Mutations in the *LDLR *gene are the most frequent cause of Familial Hypercholesterolemia (FH; Mendelian Inheritance in Man (MIM) #143890), an autosomal dominant condition characterized by elevated concentrations of LDL in blood plasma. The condition increases the risk of premature coronary artery disease due to atherosclerosis, and it occurs in about 1 in 500 in most populations [1]. The mutational spectrum is very heterogeneous in the Danish and other populations [2,3], including missense mutations, splice site mutations and large deletions [4]. Major structural rearrangements are usually considered to account for approximately 5% of mutations [5,6].

Complex rearrangements can be detected by labour-intensive techniques such as Southern blotting followed by hybridisation of labelled gene specific probes [7,8], a technique that requires large amounts of high quality DNA. We recently showed that Multiplex Ligation-Dependent Probe Amplification (MLPA) [9], which is based on semi-quantitative PCR, is a precise and effective diagnostic method [10] that makes it feasible to screen large numbers of patients for mutations of this kind.

Breakpoints have been studied in only a small number of complex rearrangements, limiting the development of diagnostic assays to detect specific deletion or duplication mutations. Ten different *LDLR *deletions have been identified in Danish FH patients [2,10-12], and the aim of this study was to characterize five deletions identified by MLPA [10] in order to define their exact extent and the breakpoints of the deletions. The results define the breakpoints of each deletion, and they suggest that unequal homologous recombination due to *Alu *repeats is responsible for the deletions. We give an example of the development of an assay that reproducibly detects the exon 7 – 8 deletion.

## Methods

### DNA

Genomic DNA was prepared from EDTA-stabilised blood samples using the PUREGENE Genomic DNA Purification Kit (Gentra Systems). The study was approved by the regional ethical committee of Aarhus County. All patients received informed consent.

### Primers and PCR

To characterize the precise locations of the genomic breakpoints, we performed a number of amplifications, using primers located in nearby exons or in bordering introns. We designed primers based on the genomic sequence extracted from the Human Genome Browser [13] using the *LDLR *reference Sequence NM_00527 (May 2004 assembly). PCR was performed using the Expand 20 kb^PLUS ^PCR system (Roche) following the manufacturer's instructions, except in case of the exon 5 deletion in which we used the Phusion High-Fidelity DNA Polymerase (Finnzymes). Primer sequences and annealing temperatures for PCR are shown in Table 1.

**Table 1:** Primers for PCR and sequencing

Deletion	PCR primers	Fragment size*	Annealing temperature	Sequencing primers
Promoter – exon1	F: gtccgaggaaggtcacagaa R: cagcacacaaatgaggtggt	3.5 kb	60	R: cagcacacaaatgaggtggt
Exon5	F: gtggtctcggccatccatcc R: tctgcaagccgcctgcaccg	1.3 kb	72**	R: tctgcaagccgcctgcaccg
Exon7 – 8	F: tcctccttcctctctctggc R: gctgcaggcaggggcgacgc	3 kb	63	R: aaagccaggcacggtggctc
Exon9 – 14	F: ggctacaagtgccagtgtga R: agctgacctttagcctgacg	2 kb	59	F: tttttgagacagagtctca R: aaagtccaaaatcaggcc
Exon 13 – 15	F: tctccttatccacttgtgtgtctag R: gctttggtcttctctgtctttgaat	8 kb	58	F: tagccaggtgtggtggcagg R: ctgggagtagctaggactgc

F: forward primer, R: reverse primer

* Fragment size for PCR fragment of the mutant allele

** Two-step PCR, 72°C 1:30 min; 98°C 10sec

PCR products were analysed in 1% agarose gels together with a normal control sample to confirm amplification of the expected allele harbouring the deletion of interest. The relevant PCR fragments were then isolated from the gel, using the QIAquick gel extraction kit (Qiagen), according to the manufacturer's instructions, or they were used directly for further analysis, if there was only one distinct band. In case of the exon 13 – 15 deletion, PCR was performed using primers amplifying "fragment 5" as described by others [14] followed by nested PCR using the isolated amplification product of 8 kilobases as template.

### Sequencing

Sequencing was performed using BigDye terminator chemistry v. 1.1 (Applied Biosystems), using a cycle sequencing protocol as recommended by the manufacturer. Sequencing reactions was ethanol precipitated, resuspended in HiDi formamide (Applied Biosystems) and separated on an Applied Biosystems 3130 Genetic Analyzer or an ABI PRISM 377 Genetic Analyzer (Applied Biosystems). PCR products were initially sequenced with the primers used for PCR and later with newly designed primers in order to obtain the sequence of the breakpoint.

### Deletion exon 7 – 8 assay

Duplex PCR to identify the exon 7–8 deletion was performed with specific primers flanking the deletion breakpoints: forward: 5'-gaaggcagtggcaagttttc-3' and reverse: 5'-gtcgatggaaccaagagtgc-3'. In the presence of one mutated allele, this PCR would normally result in a fragment of approximately 1.6 kb representing the deletion. To detect the wildtype allele we added primers amplifying DNA contained within the deletion breakpoint, thus amplifying only the wildtype allele, using the following primers: forward: 5'-ggcgaagggatgggtagggg-3' and reverse: 5'-caccactgctgcctgcaagg. The size of the wildtype fragment is approximately 1.1 kb. Both primer sets were run in one reaction, using Phusion High-Fidelity DNA Polymerase (Finnzymes) following the manufacturer's instructions. Cycling conditions were as follows: initial denaturation at 98°C for 30 seconds, followed by 30 cycles of: 10s at 98°C, 30s at 65°C and 45s at 72°C and, finally, one cycle of 7 min at 72°C.

### Bioinformatic analysis

Sequence traces were aligned to the genomic sequence of the *LDLR *gene using SeqScape version 2.5 (Applied Biosystems). The genomic sequence used as reference was taken from the May 2004 assembly in the Human Genome Browser [13] using the *LDLR *reference sequence NM_00527.

For each deletion breakpoint, the neighbouring 150 nucleotides (on each side of the breakpoint) were used to identify interspersed repeats using RepeatMasker [15], and they were aligned to the consensus *ALU *sequence described by Deininger and colleagues [16]. The exact location of the breakpoints was defined using the BLAT function [13] in the Human Genome Browser, by searching the May 2004 assembly using the sequences obtained from the amplification products harbouring the deletion of interest. Comparison of the *LDLR *sequence to a 15 bp insertion was performed using BLAST 2 sequences [17].

## Results

The data given in Table 2 show that all five probands, each having one of these deletions, have the characteristic biochemical features of FH, i.e. elevated LDL cholesterol, elevated total cholesterol, and normotriglyceridemia.

**Table 2:** Clinical characteristics of index patients with deletions in the *LDLR *gene

Deletion	Total cholesterol (mmol/l)	LDL cholesterol (mmol/l)	Triglycerides (mmol/l)
Promoter – exon1	13.0	10.4	1.7
exon 5	10.6	9.1	2.3
exon 7 – exon 8	16.0	13.9	1.5
Exon 9 – exon 14	9.1	NA	NA
Exon 13 – exon 15	8.9	7.3	1.5

Values are means of two measurements when available. NA: data not available

### Genomic characterization

**Figure 1:**
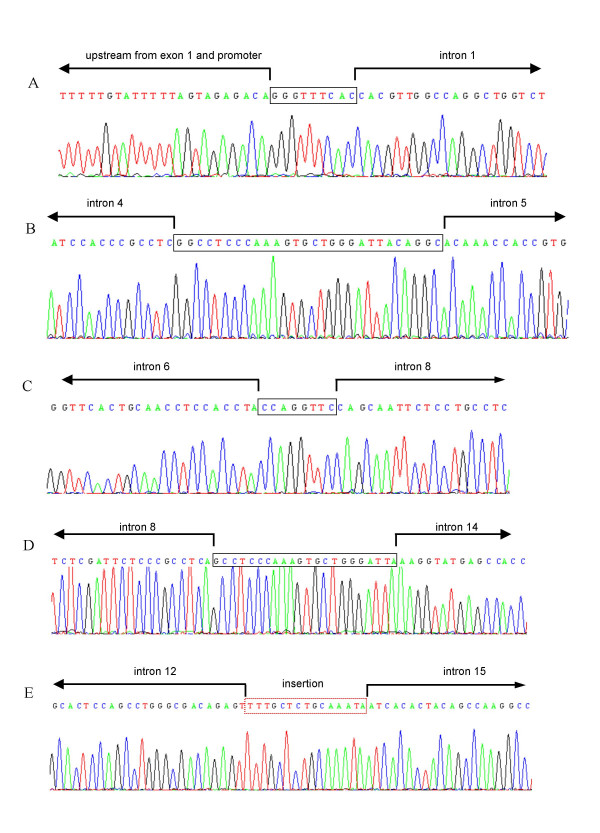
Sequence data of deletions in the *LDLR *gene. Boxed sequence represents sequence overlap between the 5' end and the 3' end of the reference sequence. Arrows represent the limits of the normal intronic sequence on each side of the breakpoint. A) Promoter – exon 1 deletion. B) Exon 5 deletion. C) Exon 7 – 8 deletion. D) Exon 9 – 14 deletion and E) Exon 13 – 15 deletion, in which the dotted red box indicates a 15 basepair insertion.

Primers and annealing temperatures used for PCR are listed in Table 1, together with approximate fragment sizes of the mutant PCR fragments and sequencing primers used to identify the breakpoints. The sequences of breakpoints and immediate flanking regions are shown in Figure 1.

### Promoter – exon 1 deletion

Initially, we used primers described by Simard et al. [18] to test whether the deletion involving exon 1 was the same as the del >15 kb described in the French-Canadian population, and if this was not the case, to determine how much of the promoter region of the gene was deleted. PCR amplification did not result in fragments of the expected size, and primers were therefore designed for an extended series of amplification reactions. One amplification resulted in a fragment of approximately 3.5 kb. This fragment was sequenced directly using new internal primers. The deletion breakpoint revealed a segment of 9 identical bases in the upstream breakpoint and the downstream breakpoint (see Figure 1), and was surrounded by a high degree of sequence similarity (data not shown). The breakpoint was flanked by *Alu *repeats (Table 3).

**Table 3:** Genomic characteristics of deletion breakpoints in the *LDLR *gene

Deletion	Deletion size, bp	5' breakpoint	3' breakpoint	Repetitive element 5'	Repetitive element 3'
Promoter – exon1	9325	11054981	11064306	*Alu *Y	*Alu *Sq
Exon5	1042	11077854	11078895	*Alu *Sx	*Alu *Jo
Exon7 – 8	3012	11081462	11084473	*Alu *Sg/x	*Alu *Sg/x
Exon9 – 14	9713	11084618	11094330	*Alu *Sq	*Alu *Sq
Exon 13 – 15	6298	11090883	11097180	*Alu *Sg/X	*Alu *Sg/x

Breakpoint locations are based on the May 2004 assembly extracted from the Human Genome Browser [13]

### Exon 5 deletion

Primers in exon 4 and exon 6 were used to amplify the deletion affecting exon 5. The resulting fragment of approximately 1.3 kb was sequenced with the reverse primer used for PCR and revealed the deletion breakpoint. Sequence data were compared to data concerning an exon 5 deletion in a Danish FH patient that we published several years ago [11]. The comparison indicated that the two deletions were the same.

### Exon 7 – 8 deletion

**Figure 2:**
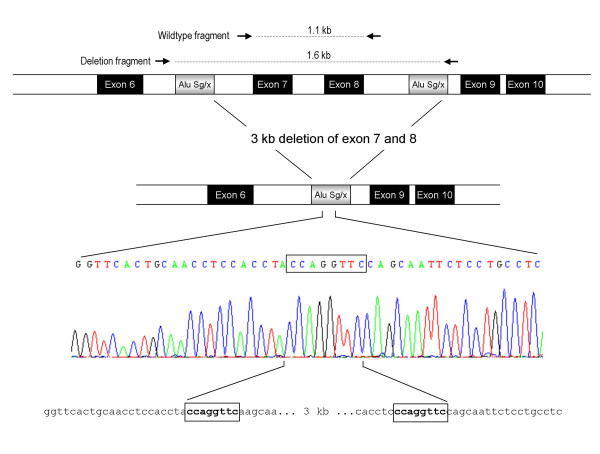
Schematic representation of the mutation deleting exon 7 and 8 of the *LDLR *gene. The locations of the primers used for the diagnostic duplex PCR is shown. Deletion breakpoints are flanked by *Alu *Sg/x elements (grey boxes). The box in the sequence data represents nucleotides present in each end of the breakpoint of the reference sequence shown below the sequence trace.

PCR with primers located in exon 6 and exon 9 revealed a PCR product of 3 kb. Sequence analysis using a primer in intron 8 revealed a breakpoint with an 8 bp overlap. The sequence flanking the breakpoint was classified as *Alu *Sg/x in both the 5' and 3' end, with a high degree of sequence identity. A schematic representation of the deletion is given in Figure 2.

### Exon 9 – 14 deletion

The mutated allele, predicted to delete 6 exons, was isolated from a PCR fragment of approximately 2 kb. The breakpoint was revealed using two sequencing primers, one located in intron 8 and one located in intron 14. It was flanked by *Alu *Sq repeats, and the overlapping sequence represents the major part of the *ALU*-DEIN consensus sequence proposed to be a recombinogenic hotspot [19].

### Exon 13 – 15 deletion

Using the method described by Kim et al [14], we amplified a fragment of 8 kb. Sequencing of a PCR fragment, generated by nested PCR using the sequencing primers (Table 1), resulted in bi-directional sequence data showing that the deletion of 6.3 kb was accompanied by an insertion of 15 bp (Figure 1). The deletion was flanked by two *Alu *Sg/x elements, and the short insertion did not show similarity to any interspersed repeats or low complexity DNA sequences, or to any other DNA sequence in the *LDLR *gene.

### Diagnostic assay for the exon 7–8 deletion

**Figure 3:**
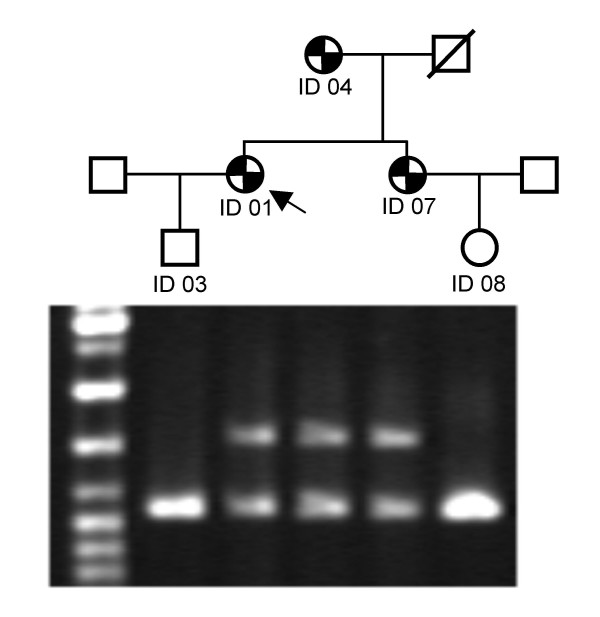
Assay to detect the exon 7 – 8 deletion. The pedigree illustrates the five individuals available (ID03, ID01, ID04, ID07 and ID08). Symbols with two black triangles represent individuals with hyperlipidemia and coronary artery disease (CAD), while open symbols represent individuals with normal lipid values and no CAD symptoms. The index patient ID01 is marked with an arrow. The left lane of the gel is a size marker, while the following lanes are the result of the duplex PCR of the individual above. In the normal individuals ID03 and ID08, only one fragment of 1.1 kb is amplified, indicating homozygosity for the normal allele. In the affected individiuals ID01, ID04 and ID07 two fragments of 1.6 and 1.1 kb is amplified, indicating heterozygosity for the mutation represented by the 1.6 kb fragment.

In order to be able to discriminate the two alleles in a family segregation for the exon 7 – 8 deletion, an assay was developed, based on duplex PCR amplifying the deletion allele, and the wildtype allele, by using two different primer pairs giving amplification products of similar size to ensure reproducible detection of both alleles. The location of the primers is shown in figure 2. As evident from Figure 3, the assay detected both the deletion and the wildtype allele in three hyperlipidemic subjects, classifying them as heterozygotes, while in two normal subjects only the wildtype specific fragment of 1.1 kb was amplified, showing that these were homozygous wildtypes.

## Discussion

In this work we describe the breakpoints of five deletions found in a Danish sample of FH patients, in which SSCP and sequencing failed to detect mutations in the *LDLR *gene [10]. The proportion of FH cases caused by large rearrangements has been estimated to be approximately 5% [5]. The number of large rearrangements reported to the FH website [4] was 92, however, accounting for 13.5% of the mutations in the database. The five deletions described in this paper account for 3.1% of the mutations identified in Danish FH mutation carriers [10], whereas a recent study of a Norwegian population of FH patients show that 10.2% of them carry complex rearrangements due to deletions or duplications in the *LDLR *[20]. A Canadian study detected 17% deletions in a sample of 70 FH patients [21]. Complex rearrangements thus account for a small but possibly quite variable proportion of LDLR mutations in different populations.

The MLPA method initially used to identify the deletions described here does not include the promoter, exon 10 or exon 13 of the *LDLR *gene. Rearrangements involving only exon 10, exon 13 or the promoter will therefore not be diagnosed using MLPA. We expanded the findings made by MLPA in the two cases (del prom – exon 1 and del 13 – 15 respectively) in which it was unclear whether the deletion included parts of the promoter or exon 13. We found that the exon 1 deletion included a large part of the sequence upstream of exon 1, including the promoter, most probably rendering this a null allele. In the case of the deletion involving exon 14 and 15, the results show that exon 13 was also deleted. A new version of the MLPA kit now includes exon 10 and 13, making findings in these regions of the gene more reliable. On the other hand, if the MLPA method detects a mutation causing only a deletion of one exon, it could possibly be a false positive result, due to the risk of failure to bind or inefficient binding of a single probe to the sample DNA. MLPA results indicating a one-exon deletion should therefore always be confirmed by an independent method.

Three other deletions involving the promoter and exon 1 have been identified previously [4], one of which was characterized recently by Canadian researchers [18]. We show that the deletion found in our patient was not identical to the one found in Canada. The deletion that we found was located approximately 6.1 kb upstream and 3 kb downstream of exon 1, whereas the French-Canadian deletion was located 11.7 kb upstream and 4 kb downstream of exon 1.

Three rearrangements deleting only exon 5 have been described earlier [8,11,22], two of them apparently smaller than the one described here. We found that the exon 5 deletion was identical to the one we have described earlier [11]. Deletions involving exon 7 – 8 and exon 13 – 15 have also been seen in other populations [4], but lack of information on precise breakpoints makes it impossible to establish whether the deletions we describe are identical to the ones reported from other populations. Mutations deleting exon 9 to 14 have to our knowledge not been identified in other populations. The diagnostic assay set up to detect the exon 7 – 8 deletion (Figure 3) proved to be a valuable and simple means of detecting affected family members. Instead of developing an assay based on ordinary long range PCR, e.g. detecting a fragment of 4.6 kb representing the wildtype allele and a fragment of 1.6 kb representing the mutated allele, we used duplex PCR, with one set of primers outside the breakpoint detecting the mutated allele, and another set of primers within the deletion breakpoints detecting the wildtype allele, to get amplification products of similar size. We were therefore able to run the PCR under relatively stringent conditions to get clear and reproducible bands from both the wildtype and mutated allele.

Since the first deletions in the *LDLR *gene were described in 1985 [7,23] it has been widely accepted that complex rearrangements can be ascribed to an abundance of *Alu *repeats in the gene, since unequal homologous recombination occurs fairly easily between two *Alu *elements, typically located in different introns [24]. *Alu *sequences represent as much as 85% of the intronic sequences of the LDLR gene [25]. The five deletions described in this work are all flanked by *Alu *elements, supporting a mutation mechanism involving unequal homologous recombination between highly similar *Alu *elements. Rüdiger and colleagues [19] suggested that a 26 basepair part of an *Alu *consensus sequence (*ALU*-DEIN) served as a recombinogenic hotspot, not only because it occurs close to several complex rearrangements in the *LDLR *and other genes, but also because it contains a motif known to mediate recombination in *Escherichia coli*. The *ALU*-DEIN sequence was also present in the exon 5 deletion identical to the one described here; it occurs close to the 5' flank of the promoter – exon 1 deletion; and it constitutes the major part of the overlapping sequence in the exon 9 – 14 deletion. Recently the hypothetical recombinogenic hotspot was also suggested to explain the occurrence of two deletions in the *ALD *gene [26], lending further support to the hypothesis that the *ALU*-DEIN sequence is a recombinogenic hotspot that can cause unequal homologous recombination.

## Conclusion

In conclusion, the present study defines the precise genomic breakpoints of five deletions in the *LDLR *gene, opening new possibilities to develop specific diagnostic tests for these deletions. Furthermore, it supports the proposition that *Alu *repeats are involved in mutational events in the *LDLR*.

## Competing interests

The author(s) declare that they have no competing interests.

## Authors' contributions

PHN designed the study, performed bioinformatic studies, participated in the interpretation of the data and drafted the manuscript. DD participated in the design of the study and helped to draft the manuscript. AS and GGN carried out molecular genetic studies and participated in the interpretation of the data. OF selected and evaluated patients and helped to draft the manuscript. MLN selected and evaluated patients. All authors read and approved the final manuscript.

## Pre-publication history

The pre-publication history for this paper can be accessed here:


